# A Novel Method for Determining Fibrin/Fibrinogen Degradation Products and Fibrinogen Threshold Criteria via Artificial Intelligence in Massive Hemorrhage during Delivery with Hematuria

**DOI:** 10.3390/jcm13061826

**Published:** 2024-03-21

**Authors:** Yasunari Miyagi, Katsuhiko Tada, Ichiro Yasuhi, Keisuke Tsumura, Yuka Maegawa, Norifumi Tanaka, Tomoya Mizunoe, Ikuko Emoto, Kazuhisa Maeda, Kosuke Kawakami, on behalf of the Collaborative Research in National Hospital Organization Network Pediatric and Perinatal Group

**Affiliations:** 1 Medical Data Labo, 289-48 Yamasaki, Naka Ward, Okayama 703-8267, Japan; 2 Miyake Ofuku Clinic, 393-1 Ofuku, Minami Ward, Okayama 701-0204, Japan; 3 Department of Obstetrics and Gynecology, NHO Okayama Medical Center, 1711-1 Tamasu, Kita Ward, Okayama 701-1192, Japan; ktada1221@gmail.com; 4 Department of Obstetrics and Gynecology, NHO Nagasaki Medical Center, 2-1001-1 Kubara, Omura 856-8562, Japan; iyasuhi0414@gmail.com; 5 Department of Obstetrics and Gynecology, NHO Saga Hospital, 1-20-1 Hinode, Saga 849-8577, Japan; tmrksk.obgy@gmail.com; 6 Department of Obstetrics and Gynecology, NHO Mie Chuo Medical Center, 2158-5 Hisaimyojincho, Tsu 514-1101, Japan; yuka.ma0513@gmail.com; 7 Department of Obstetrics and Gynecology, NHO Higashihiroshima Medical Center, 513 Saijochojike, Higashihiroshima 739-0041, Japan; tanaka.norifumi.an@mail.hosp.go.jp; 8 Department of Obstetrics and Gynecology, NHO Kure Medical Center, 3-1 Aoyama, Kure 737-0023, Japan; mizunoe.tomoya.fd@mail.hosp.go.jp; 9 Department of Obstetrics and Gynecology, NHO Kyoto Medical Center, 1-1 Fukasakumukaihata, Kyoto 612-8555, Japan; emoto.ikuko.yh@mail.hosp.go.jp; 10 Department of Obstetrics and Gynecology, NHO Shikoku Medical Center for Children and Adults, 2-1-1 Senyucho, Zentsuji 765-8507, Japan; maeda.kazuhisa.hf@mail.hosp.go.jp; 11 Department of Obstetrics and Gynecology, NHO Kokura Medical Center, 10-1 Harugaoka, Kokuraminami Ward, Kitakyushu 802-8533, Japan; kawakami.kosuke.ed@mail.hosp.go.jp

**Keywords:** artificial intelligence, delivery, DIC, hemorrhage, machine learning

## Abstract

**(1) Background:** Although the diagnostic criteria for massive hemorrhage with organ dysfunction, such as disseminated intravascular coagulation associated with delivery, have been empirically established based on clinical findings, strict logic has yet to be used to establish numerical criteria. **(2) Methods:** A dataset of 107 deliveries with >2000 mL of blood loss, among 13,368 deliveries, was obtained from nine national perinatal centers in Japan between 2020 and 2023. Twenty-three patients had fibrinogen levels <170 mg/dL, which is the initiation of coagulation system failure, according to our previous reports. Three of these patients had hematuria. We used six machine learning methods to identify the borderline criteria dividing the fibrinogen/fibrin/fibrinogen degradation product (FDP) planes, using 15 coagulation fibrinolytic factors. **(3) Results:** The boundaries of hematuria development on a two-dimensional plane of fibrinogen and FDP were obtained. A positive FDP–fibrinogen/3–60 (mg/dL) value indicates hematuria; otherwise, the case is nonhematuria, as demonstrated by the support vector machine method that seemed the most appropriate. **(4) Conclusions:** Using artificial intelligence, the borderline criterion was obtained, which divides the fibrinogen/FDP plane for patients with hematuria that could be considered organ dysfunction in massive hemorrhage during delivery; this method appears to be useful.

## 1. Introduction

Massive hemorrhage continues to be one of the most severe complications for pregnant women. Massive hemorrhage during delivery can be classified into consumption coagulopathy with coagulation abnormalities, such as disseminated intravascular coagulation (DIC) resulting from abruptio placentae, and dilutional coagulopathy without coagulation abnormalities, such as atonic bleeding [1]. DIC is a systemic disease affecting the coagulation system, simultaneously causing procoagulant factor activation, fibrinolytic activation, and consumptive coagulopathy, which can ultimately lead to organ dysfunction and death [2]. As DIC is a cause of maternal mortality, the pathogenesis of the disease needs to be understood. DIC should be appropriately diagnosed because massive hemorrhage during delivery due to consumption coagulopathy may require treatment for correction of coagulation abnormalities. DIC during pregnancy is one of the chief causes of maternal mortality worldwide [3,4], with a frequency of 0.03% [5] to 0.35% [6]. DIC can originate from and cause damage to the microvasculature; this, if sufficiently severe, can lead to organ dysfunction [7]. However, although the numerical diagnostic criteria for DIC associated with delivery have been empirically established based on clinical findings such as the presence or absence of organ dysfunction and laboratory findings of coagulation and fibrinolysis factors, strict logic has yet to be used to establish numerical criteria.

Fibrin/fibrinogen degradation products (FDPs) represent a key factor concerning DIC. An excessive FDP level with diminished or normal systemic fibrinolytic activity suggests that local intravascular fibrin deposition and fibrinolysis occur in normal parturition and bleeding complications in pregnancy [8]. We previously reported the criterion values for coagulation and fibrinolytic system collapse based on FDPs, focusing on the fibrinogen behavior, using mathematical methods such as data distribution function evaluation and differential equations, in massive hemorrhage during delivery without organ dysfunction [9]. When the fibrinogen level fell below 237 mg/dL, the FDP distribution became abnormal, which began the abnormal coagulation/fibrinolysis system. When the fibrinogen level fell below 170 mg/dL, the coagulation/fibrinolysis system failed (*p* < 0.05). The borderline FDP value in organ dysfunction cases was expected to be higher. This classification showed good agreement with the clinical status [10].

Artificial intelligence (AI) has recently become a more common and easily applied tool in medical science. Some AI applications involve medical imaging [11,12,13,14,15], though some do not [16,17,18,19]. Machine learning is part of the concept of AI and can be used to acquire rules for judging unknown data by learning the latent patterns in the data. Machine learning enables the learning and classification of observed data. An optimal method can be chosen from among various machine learning algorithms. Machine learning can be used to computationally classify data, thereby determining the data boundaries.

This was a retrospective study in which we collected cases of massive hemorrhage during delivery with hematuria that could be assumed to be organ dysfunction, and we developed a method to mathematically determine the FDP boundaries for hematuria.

We used the method to analyze 15 coagulation/fibrinolysis-related factors, selecting factors that correlate with fibrinogen and FDP, facilitate rapid clinical results, and prevent multicollinearity with each other. Then, after performing supervised learning using machine learning, we determined the criterion line dividing the plane of fibrinogen and FDP. We used the same selected factors to create a discriminant analysis function [20] to obtain the boundaries, which we compared with the boundaries created by machine learning.

## 2. Materials and Methods

This multicenter retrospective case series study, including human subjects, was approved by the National Hospital Organization (NHO) Central Research Ethics Committee (R1-1009002). The participants were women who gave birth at any of the nine NHO perinatal centers (NHO Okayama Medical Center, NHO Saga Hospital, NHO Nagasaki Medical Center, NHO Mie Chuo Medical Center, NHO Higashihiroshima Medical Center, NHO Kure Medical Center, NHO Kyoto Medical Center, NHO Shikoku Medical Center for Children and Adults, and NHO Kokura Medical Center) in Japan between August 2020 and September 2023. The inclusion criteria for the study were as follows: (1) women with >2000 mL of blood loss within 24 h of delivery; (2) any mode of delivery, either vaginal or cesarean; (2) singleton or multiple pregnancies; and (3) delivery after 22 weeks of gestation. The rationale for inclusion criteria (1) and (2) was as follows: coagulopathy rarely develops when blood loss is ≤2000 mL [21]. Furthermore, women who had bleeding of > 2000 mL at delivery, regardless of mode of delivery, are defined by the Royal College of Obstetrics and Gynaecologists as having severe PPH [22]. The exclusion criteria included (1) women with medical complications that could cause coagulopathy and (2) women taking aspirin or other medications that affect the coagulation system.

Blood loss was evaluated using different methods. During vaginal delivery, blood loss was weighed by subtracting the dry weight of the absorbent pad from the weight of the blood-soaked pad and/or from direct blood collection. All blood lost was contained in a collector bag, which was placed under the woman’s buttocks. During cesarean delivery, dry and blood-soaked surgical pads were weighed before and after surgery, and/or blood volume aspirated from the surgical field with a suction tube was determined in milliliters. The units of measured blood loss were standardized to milliliters, as grams are known to be almost equivalent to milliliters in weight.

The attending physicians obtained blood samples when deemed clinically necessary, regardless of blood loss. Activated partial thromboplastin time (APTT), D-dimer, FDP, fibrinogen, hematocrit (Hct), hemoglobin (Hgb), platelet (Plt), prothrombin time (PT-sec) [6], and prothrombin time-international normalized ratio (PT-INR) were measured in each center’s laboratory for clinical management. Antiplasmin (AP), antithrombin (AT), fibrin monomer complex (FMC), α2 plasmin inhibitor plasmin complex (PIC) [23], and thrombin antithrombin complex (TAT) [23] levels were also measured in the laboratories of SRL INC. (Tokyo, Japan) for this study. These 14 blood test indicators analyzed in the study were used as factors for the complete data. We used fully deidentified data in this study.

Obstetric management, blood product transfusion, and determining the cause of bleeding were performed at the attending physicians’ discretion at each center. All nine centers participating in the study were perinatal centers in Japan, which routinely managed high-risk pregnancies, including those with severe PPH, which would have allowed for management without substantial differences. Data from the first measurement for each patient were used for analysis.

All procedures were performed in accordance with the ethical standards of the committees responsible for human experimentation (institutional and national) and the Declaration of Helsinki of 1964 and its later amendments. Written informed consent was obtained from all patients for whom identifying information is included in this article.

Based on the results of our previous study [9], we used data for fibrinogen <170 mg/dL only, which was a criterion for coagulation system failure. In this study, we defined the appearance of hematuria as organ dysfunction [24].

Let the set of factors of the complete data obtained be {*α*, *β*, *γ*, …}. Let {*α*, *β*, *γ*, …} be the set of factors of the complete data obtained. Let *A* and *B* be the sets of factors significantly related to element *α* and another element *β*, respectively. *A* contribution rate > 0.49 is used as the criterion. The elements of the *A* ∪ *B* set are classified into the following sets: *P*, *Q*, and *R*.

(1) $$P=\left\{x|x\in A,x\notin B,x\notin \Phi \right\}$$

(2) $$Q=\left\{y|y\notin A,y\in B,y\notin \Phi \right\}$$

(3) $$R=\left\{z|z\in A,z\in B,z\notin \Phi \right\}$$

where *cr*(*v*, *w*) is the contribution rate between *v* and *w*. Then,

(4) $$cr\left(x_{s},x_{t}\right)<CR;\;s\neq t,\;\forall x_{s}\in P,\;{\forall x}_{t}\in P$$

(5) $$cr\left(y_{s},y_{t}\right)<CR;\;s\neq t,\;{\forall y}_{s}\in Q,\;\forall y_{t}\in Q$$

(6) $$cr\left(z_{s},z_{t}\right)<CR;\;s\neq t,\;\forall z_{s}\in R,\;\forall z_{t}\in R$$

Here, *CR* = 0.64. When *N*(*P*), *N*(*Q*), and *N*(*R*) are the number of elements in *P*, *Q*, and *R*, respectively, we have the following vectors:

(7) $$\alpha ,\;\beta ,{\;x}_{1},{\;x}_{2},\ldots ,x_{N\left(P\right)},\;{\;y}_{1},{\;y}_{2},\ldots ,y_{N\left(Q\right)},\;z_{1},\;z_{2},\ldots ,z_{N(R)}$$

The classifiers were created by labeling cases with and without organ dysfunction and performing supervised machine learning. As machine learning methods, we used logistic regression, which is a widely used method for predicting binary outcomes and can be used for multiple outcomes [25,26]; random forest, which is a popular supervised method in which an ensemble approach is employed by combining multiple decision trees through the bagging technique to increase the accuracy and robustness of the model [26,27]; nearest neighbors, which is a nonparametric supervised learning method that produces the output classified by a plurality vote of its neighbors, with the object being assigned to the class most common among its nearest neighbors [28,29]; naïve Bayes, which is optimal when attributes are independent given the class [30,31]; neural network, which is a branch of machine learning models inspired by the neuronal organization found in the biological neural networks in animal brains [32,33]; and support vector machine, which is a machine learning algorithm that can be used for both linear and nonlinear classification tasks and constructing hyperplanes in a high dimensional space [26,34].

When the regression functions are *f*, *g*, and *h*,

(8) $${\tilde{x}}_{i}=f_{i}\left(\alpha \right);\;i=\{1,\;2,\;\ldots ,\;N\left(P\right)\}$$

(9) $${\tilde{y}}_{j}=g_{j}\left(\beta \right);\;j=\{1,\;2,\;\ldots ,\;N\left(Q\right)\}$$

(10) $${\tilde{z}}_{k}=h_{k}\left(\alpha ,\beta \right);\;k=\{1,\;2,\;\ldots ,\;N\left(R\right)\}$$

(11) $$\alpha ,\;\beta ,{\;\tilde{x}}_{1},{\;\tilde{x}}_{2},\ldots ,{\tilde{x}}_{N\left(P\right)},\;{\tilde{y}}_{1},{\;\tilde{y}}_{2},\ldots ,{\tilde{y}}_{N\left(Q\right)},\;{\tilde{z}}_{1},\;{\tilde{z}}_{2},\ldots ,{\tilde{z}}_{N(R)}$$

This vector was substituted into the classifier to obtain the classification in {*α*, *β*}. In this study, *α* and *β* were calculated as fibrinogen and FDP, respectively. Next, a discriminant analysis was performed using this vector. Then, a single regression function $\tilde{\alpha }=f\left(\beta \right)$ was obtained using the same method, with fibrinogen as the dependent variable and FDP as the independent variable. The organ dysfunction criterion values for FDP alone were then determined.

We examined the performance of each machine learning method, and we comprehensively determined the optimal boundary criterion.

Development environment for analysis and statistics:

The computing environment comprised a Mac Pro running OS X 13.1 (Apple Inc.; Cupertino, CA, USA) and Mathematica 13.0 (Wolfram Research; Champaign, IL, USA). The data distribution analysis, discriminant analysis, linear regression analysis, machine learning, Mann–Whitney test, and *t*-test were conducted with this setup.

## 3. Results

During the study period, 322 women fulfilled the inclusion criteria from among 13,368 deliveries at all participating centers. Of these women, 190 were unable to send blood samples to SRL INC. For the study, 132 were enrolled, and 25 were excluded because some required data were missing. In this study’s final sample of 107 women, 14 (13%) had already been administered red blood cell concentrate (RCC) and/or fresh–frozen plasma (FFP) (8 of both, 5 of RCC only, and 1 of FFP only) at the time of first blood sampling. Of the 107 women, 23 had a fibrinogen level < 170 mg/dL, and 8 (35%) were administered RCC and/or FFP (7 of both and 1 of FFP only). In 23 of the women with a fibrinogen level < 170 mg/dL, 3 were classified as organ dysfunction cases because they had hematuria corresponding to marked activation of the coagulation–fibrinolytic system, whereas the remaining 20 women were non-organ-dysfunction cases. The 15 factors, that is, the 14 blood test parameters described in the Methods section plus the Hgb/fibrinogen ratio, were obtained as factors for the complete data. Among these 15 factors, those with a significant difference between patients with and without organ dysfunction were FDP, D-dimer, TAT, Hgb/fibrinogen, fibrinogen, PT-sec, and PT-INR in the *t*-test, and FDP, D-dimer, TAT, Hgb/fibrinogen, fibrinogen, PT-INR, PT-sec, and PIC in the Mann–Whitney test in descending order of *p*-value from the *t*-test (Table 1). In both tests, FDP was the most statistically significantly different.

Among the 13 factors other than fibrinogen and FDP, Hgb/fibrinogen, PT-sec, and PT-INR strongly correlated with fibrinogen, whereas D-dimer and PIC strongly correlated with FDP. To avoid multicollinearity, regression functions were created to obtain Hgb/fibrinogen and PT-sec from fibrinogen and D-dimer from FDP (Table 2).

**Figure 1:**
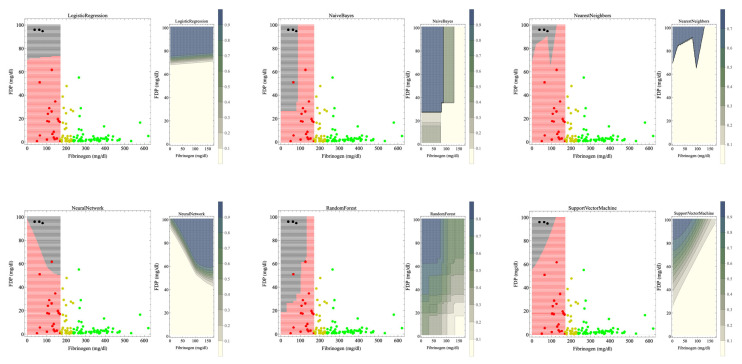
Boundary of hematuria occurrence in heavy bleeding during delivery determined using machine learning, i.e., the boundary of organ dysfunction occurrence. The results of each machine learning method are shown in two sets of figures. The left figure of the set shows the scatterplot of the data and the boundaries of the hematuria occurrence. The black areas indicate areas of organ dysfunction. The green, yellow, red, and black dots indicate cases without coagulopathy, with coagulopathy, with disrupted coagulopathy, and with organ dysfunction, respectively. In our previous study, we used fibrinogen criteria values of 237 and 170 mg/dL for the development and disruption of coagulopathy, respectively [9]. The machine learning classifier was trained on red and black cases with fibrinogen levels < 170 mg/dL. The crude areas where organ dysfunction occurred for each method were as follows: logistic regression, FDP > 75 mg/dL; naïve Bayes, fibrinogen < 100 mg/dL and FDP > 30 mg/dL; nearest neighbors, fibrinogen < 120 mg/dL and FDP > 80 mg/dL; neural network, FDP > 50 mg/dL; random forest, fibrinogen < 150 mg/dL and FDP > 40 mg/dL. For the support vector machine, FDP–fibrinogen/3–60 (mg/dL) is positive because FDP decreases from 100 to 60 mg/dL as the fibrinogen level decreases from 120 to 0 mg/dL. The right figure shows the contours of the estimated probability of hematuria occurrence, divided into 10 segments from 0% to 100%. as shown in the legend bar presenting that the darker the color, the higher the probability of hematuria. If the estimated probability of developing hematuria was >0.5, the patient was considered to have hematuria in the left figure. As coagulopathy is generally accompanied by a fibrinogen decrease and an FDP increase, support vector machines, neural networks, and naïve Bayes, the contour lines of which change stepwise, seemed to be good fits. The boundary determined using the support vector machine method that does not include the fibrinogen 170 mg boundary seemed most appropriate for clinical use.

Figure 1 shows the hematuria boundaries that, based on machine learning, were recognized as organ dysfunction in massive hemorrhage during delivery. In a previous study [9], the reference values for fibrinogen were 237 and 170 mg/dL for the onset and breakdown of coagulopathy, respectively. Machine learning was used to perform supervised learning on cases with fibrinogen levels < 170 mg/dL. The patient was considered to have hematuria if the estimated probability of developing hematuria by each machine learning method was >0.5. The crude areas where organ dysfunction occurs were as follows for the different models: logistic regression: FDP > 75 mg/dL; naïve Bayes: fibrinogen < 100 mg/dL and FDP > 30 mg/dL; nearest neighbors: fibrinogen < 120 mg/dL and FDP > 80 mg/dL; neural network: FDP > 50 mg/dL; random forest: fibrinogen < 150 mg/dL and FDP > 40 mg/dL. For the support vector machine, a positive FDP–fibrinogen/3–60 (mg/dL) value indicates hematuria; otherwise, the case is nonhematuria because FDP decreases from 100 to 60 mg/dL as the fibrinogen level decreases from 120 to 0 mg/dL.

In Figure 1 (left), for each method, the black areas indicate areas of organ dysfunction. The green, yellow, red, and black dots indicate cases without coagulopathy, with coagulopathy, with disrupted coagulopathy, and with organ dysfunction, respectively. In our previous study, we used fibrinogen criteria values of 237 and 170 mg/dL for the development and disruption of coagulopathy, respectively [9]. The machine learning classifier was trained on red and black cases with fibrinogen levels < 170 mg/dL. The crude areas where organ dysfunction occurred for each method were as follows: logistic regression, FDP > 75 mg/dL; naïve Bayes, fibrinogen < 100 mg/dL and FDP > 30 mg/dL; nearest neighbors, fibrinogen < 120 mg/dL and FDP > 80 mg/dL; neural network, FDP > 50 mg/dL; random forest, fibrinogen < 150 mg/dL and FDP > 40 mg/dL. For the support vector machine, FDP–fibrinogen/3–60 (mg/dL) is positive because FDP decreases from 100 to 60 mg/dL as the fibrinogen level decreases from 120 to 0 mg/dL.

In the contour graph in Figure 1 (right) for each method, as shown in the legend bar, the darker the color, the higher the probability of hematuria. If the estimated probability of developing hematuria was >0.5, the patient was considered to have hematuria in the left figure. As coagulopathy is generally accompanied by a fibrinogen decrease and an FDP increase, support vector machines, neural networks, and naïve Bayes, the contour lines of which change stepwise, seemed to be good fits.

The boundary determined using the support vector machine method that does not include the fibrinogen 170 mg boundary seemed most appropriate for clinical use.

Table 3 shows the classification performance on the boundaries in the fibrinogen and FDP planes of hematuria occurrence by the different machine learning methods. All methods were highly accurate (>0.91). The area under the characteristic curve (AUC) was high for all methods (>0.95). Class mean class entropy [35,36] was smaller for the logistic regression, naïve Bayes, and neural network methods.

**Figure 2:**
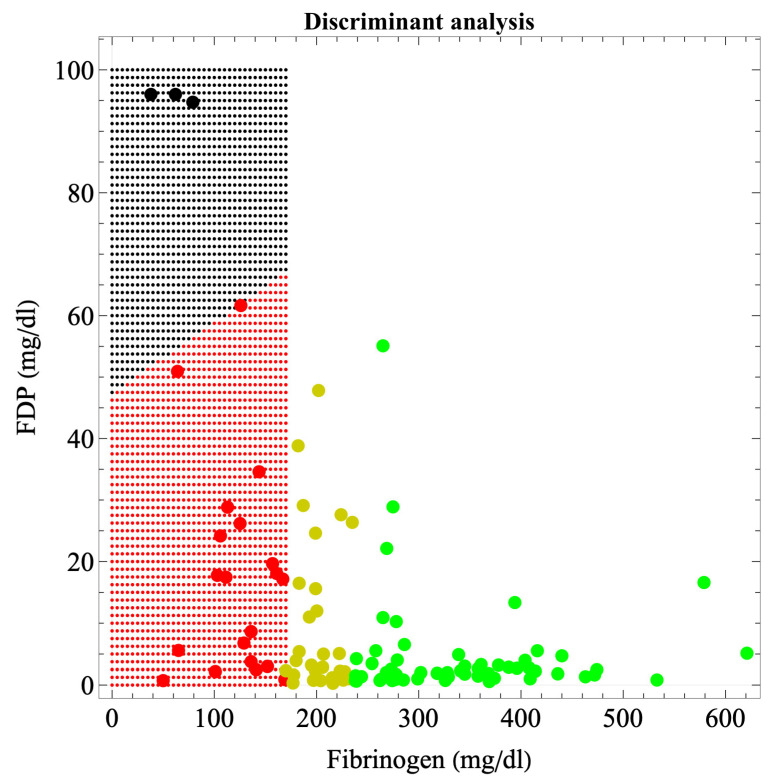
Boundary of hematuria occurrence in major hemorrhage at parturition according to discriminant analysis, i.e., the boundary of organ dysfunction occurrence. Black areas indicate areas of organ dysfunction. The green, yellow, red, and black dots indicate cases without coagulopathy, with coagulopathy, with disrupted coagulopathy, and with organ dysfunction, respectively. In our previous study, we used fibrinogen criteria values of 237 and 170 mg/dL for the development and disruption of coagulopathy, respectively [9]. The boundary was a straight line connecting (0, 50) and (170, 68) in the fibrinogen–FDP coordinates. The discriminant function was 50.693 − 0.00106 × fibrinogen − 0.52723 × FDP − 0.04314 × Hgb/fibrinogen − 0.6978 × PT-sec − 0.02844 × D-dimer; $\eta^{2}=0.775$, which indicates a moderate fit (*p* = 3.37 × 10^−9^); error probability = 0.4186%.

Figure 2 shows the boundaries of hematuria occurrence in massive hemorrhage during delivery, according to discriminant analysis. The boundary was a straight line connecting (0, 50) and (170, 68) in the fibrinogen–FDP coordinates. The discriminant function was 50.693 − 0.00106 × fibrinogen − 0.52723 × FDP − 0.04314 × Hgb/fibrinogen − 0.6978 × PT-sec − 0.02844 × D-dimer; the η^2^ of 0.775 indicated a moderate fit (*p* = 3.37 × 10^−9^), with an error probability of 0.4186%.

The FDP criterion value for hematuria development was determined from the FDP value alone, independent of fibrinogen. The relationship between fibrinogen and FDP is $\tilde{y}=\beta_{0}+\beta_{1}x$; x; FDP, y; fibrinogen, $\beta_{0}=132.905\pm 9.571\left(P=4.719\times 10^{-12}\right)$, $\beta_{1}$; $-0.6613\pm 0.2327\;\left(P=9.760\times 10^{-3}\right)$, $R^{2}=0.278$. Table 4 compares the performance of the classifiers among the machine learning methods. The FDP criteria were 84.96, 101.16, 73.38, 86.94, 92.23, and 79.67 for logistic regression, random forest, nearest neighbors, naïve Bayes, neural network, and support vector machine, respectively. All AUC values, except for that of random forest, were >0.958, and the positive diagnostic rate was excellent, at ≥0.956.

## 4. Discussion

We developed a method for determining the boundaries of hematuria development associated with massive hemorrhage during delivery on a two-dimensional plane consisting of fibrinogen and FDP in this retrospective study presented with reference to the STROBE statement that was used for cohort studies, case–control studies, and cross-sectional studies [37]. Each machine learning method and discriminant analysis produced a variety of candidate boundaries. As shown in Table 3, the performance of the machine learning classifier and the fit of the discriminant analysis function were both generally excellent. The different methods produced various boundaries and criteria; however, as they are all mathematically correct, simply determining a single boundary or a criterion statistically was undesirable, as was adopting an average of the values. Boundaries should be comprehensively determined based on clinical judgment, though data analysis should eliminate subjective judgments to the largest possible extent. As coagulopathy is generally accompanied by decreased fibrinogen and increased FDP levels, the support vector machine, neural network, and naïve Bayes methods are well suited for the task at hand because the contour lines change in a stepwise manner. Additionally, none of these three methods produced a fibrinogen 170 mg/dL boundary. Based on these findings, the support vector machine and naïve Bayes methods appeared suitable for predicting hematuria in patients with massive hemorrhage during delivery. The boundary of whether FDP–fibrinogen/3–60 (mg/dL) is positive according to the support vector machine method seemed the most appropriate for clinical use, though naïve Bayes, for which the class mean class entropy was low, may be optimal (Figure 1, Table 3). Erez et al. [38] reported that the definition of DIC by the Scientific and Standardization Committee on DIC of the International Society on Thrombosis and Haemostasis indicated that (1) DIC is always secondary to other causes, one being obstetric-related, such as abruption placentae [39]; (2) DIC represents the systemic pathological activation of coagulation; and (3) DIC is a process that originates in the microvasculature, or the vascular endothelium, resulting in organ damage from microthrombi [40]. As hematuria alone does not necessarily indicate organ dysfunction, yet is almost always considered to be nearly a state of organ dysfunction, and as biopsying microthrombi in the kidneys of pregnant women in delivery who are hemorrhaging is impractical, we need to define the onset of hematuria associated with massive hemorrhage during delivery as a type of clinical DIC and analyze and apply the findings in clinical practice.

Although many coagulation and fibrinolytic factors exist in addition to, for example, complete blood counts and platelets, in a massive hemorrhage under the urgent circumstances of delivery, factors should be selected so that only those for which test results are available as quickly as possible are used to predict organ dysfunction and initiate preventive treatment. We, therefore, sought a criterion for determining organ dysfunction based solely on fibrinogen and FDP, factors for which results are obtained relatively quickly. As information on related factors such as Hgb/fibrinogen ratio, PT-sec, and D-dimer also contribute to these factors, five-dimensional factors were projected onto a two-dimensional plane using a regression function; boundaries were not simply determined from fibrinogen and FDP information alone. We also obtained the boundaries of the FDP only in one dimension (Table 4). Clinicians would find the boundaries created from fibrinogen and FDP in a two-dimensional plane (Figure 1 and Figure 2) more clinically convincing than the one-dimensional FDP boundaries because the fibrinogen estimates from the FDP are less precise. This is because, even at fibrinogen levels of <170 mg/dL, consumption coagulopathy with FDP elevation and dilutional coagulopathy without FDP elevation were mixed; thus, the correlation between fibrinogen and FDP is no longer accurate (R^2^ = 0.278). Therefore, boundaries in a two-dimensional plane are more realistic and suitable for clinical applications than a one-dimensional criterion. Although a boundary surface in a higher-dimensional space would be more accurate, recognizing and handling a boundary surface that divides a space of more than three dimensions is challenging; therefore, a boundary in a two-dimensional plane appears to be the most appropriate. The recognition of boundary surfaces in three-dimensional space and beyond will become easier to understand if the space is subdivided, that is, if the dimension is lowered by dividing the space into cases under certain conditions.

In this case, the boundary values are obtained in a two-dimensional plane from multidimensional factors, but by extending the above-described method, boundary surfaces can be obtained in a three-dimensional or larger space. This can be not only applied to the analysis of the coagulation–fibrinolytic system in massive hemorrhage but also further generalized to other research areas. Care must be taken, however, when combining selected factors to avoid multicollinearity. No set rules exist for combining these factors; these must be handled on a case-by-case basis, which complicates the creation of the regression function. The discriminant analysis showed results comparable with those of machine learning, though this method only provides a linear combination of factors. However, because each factor is supposedly related to the others, AI classifiers are generally more suitable and will be especially so in the future. If more cases (e.g., more than 1000) can be accumulated, including many organ dysfunction cases, AI using deep learning [41,42] should provide more reliable boundaries than machine learning or discriminant analysis.

In this study, we sought the boundary between the two classifications of the presence or absence of organ dysfunction, but more than three classifications are obtainable by extending the above-described method. Our analysis was limited to fibrinogen < 170 mg/dL, where the coagulation system is disrupted, in accordance with our previous report [9], but boundaries can be created between three or more regions, including normal conditions.

The most statistically significant difference in FDP was between organ-dysfunctional and non-organ-dysfunctional cases with a fibrinogen level of <170 mg/dL. This suggests that FDP is the most important factor in determining organ dysfunction. Regarding hemorrhage, we suggest the validity of using FDP to predict severe organ dysfunction, focusing on the dynamics of fibrinogen, which is involved in both coagulation and fibrinolysis.

This study had some limitations. First, the number of organ dysfunction cases (three) was low. The results were acceptable despite having only three cases of organ dysfunction because the cases’ laboratory values were widely different and extremely abnormal. However, the frequency of detection of hematuria was 2.27% (3/132) for the cases for whom the amount of bleeding was >2000 mL at delivery and only 0.0244% (3/13, 368) of all deliveries; thus, accumulating cases of organ dysfunction such as hematuria is challenging. Ideally, more data from more organ dysfunction cases would be added to the analysis. Second, although fibrinogen and FDP were selected as the two items for which rapid test results were clinically obtainable, the ability to obtain rapid results for both at the same time depends on medical institutions’ facilities and testing methods’ advances. In facilities where only fibrinogen or FDP results are available, fibrinogen levels < 100 mg/dL or FDP levels > 60 mg/dL should be considered as an indication of organ dysfunction. Furthermore, although this is not our contention, some factors other than fibrinogen and FDP may be better suited for predicting organ dysfunction. Finally, although no deaths occurred in this study and hematuria was used as organ dysfunction, a fixed definition does not exist for organ dysfunction. Data should be analyzed with other indicators, such as death, renal dysfunction, postpartum sequelae, and histopathological abnormalities. The use of these severe cases would lead to different results.

## 5. Conclusions

Although each machine learning method proposed various boundaries, the results of naïve Bayes, support vector machines, and discriminant analysis seem clinically acceptable. The boundary for whether FDP–fibrinogen/3–60 (mg/dL) is positive according to the support vector machine seemed the most appropriate for clinical use. In the future, a more reliable region will be available if more types of organ dysfunction or data on cases of organ dysfunction can be added. Even if the definition of organ dysfunction is changed or if many factors, including unknown ones, are added to the definition of organ dysfunction, the method developed in this study, which is based on AI using multiple factors correlated with fibrinogen and FDP and avoiding multicollinearity to obtain the boundary line dividing the plane of fibrinogen and FDP, can be expected to be a useful criterion for identifying organ dysfunction occurrence.

## Figures and Tables

**Table 1:** Comparison of factors for organ- and non-organ-dysfunction cases in data with fibrinogen <170 mg/dL. Factors are listed in order of decreasing *p*-value on *t*-test.

	Organ Dysfunction (Hematuria)						Non-Organ Dysfunction						*t*-Test	Mann–Whitney Test
Factor	Mean ± SD	Min	Max	Median	0.25 Quantile	0.75 Quantile	Mean ± SD	Min	Max	Median	0.25 Quantile	0.75 Quantile	*p*-Value	*p*-Value
FDP	95.58 ± 0.73	94.73	96	96	94.73	96	17.52 ± 16.8	0.72	61.64	17.31	3.01	24.16	1.03 × 10^−7^	0.0053
DD	350.63 ± 189.25	135.4	491	425.5	135.4	491	47.77 ± 54.65	3	219	34.05	10.61	47.9	3.32 × 10^−6^	0.0092
TAT	120 ± 0.0	120	120	120	120	120	67.53 ± 42.88	15.4	120	63.25	19.9	113	2.80 × 10^−5^	0.0319
Hgb/fbg	139.08 ± 73.17	56.96	197.37	162.9	56.96	197.37	61.42 ± 24.9	30.18	112	53.96	42.64	66.67	0.0009	0.0399
fbg	59.67 ± 20.6	38	79	62	38	79	122.82 ± 34.2	50	169	127.5	103	143.8	0.0057	0.0198
PT-sec	17.3 ± 2.14	15.6	19.7	16.6	15.6	19.7	13.62 ± 2.24	10.1	18.2	13.2	12	14.4	0.0145	0.0318
PT-INR	1.61 ± 0.2	1.46	1.84	1.53	1.46	1.84	1.22 ± 0.25	0.96	2	1.11	1.06	1.22	0.0174	0.0317
PIC	38.13 ± 26.67	17.7	68.3	28.4	17.7	68.3	8.87 ± 8.02	0.3	27.3	6.45	2	14.6	0.1962	0.0121
AP	45.33 ± 11.93	32	55	49	32	55	53.7 ± 14.28	34	93	52.5	42	56	0.3479	0.4107
AT	52.67 ± 19.01	34	72	52	34	72	45.2 ± 13.15	27	82	43	39	49	0.3925	0.4364
APTT	54.3 ± 19.54	39.3	76.4	47.2	39.3	76.4	46.28 ± 17.05	29.7	93.6	40.65	32.5	51.8	0.4624	0.3858
FMC	166.33 ± 121.48	27	250	222	27	250	184.12 ± 83.87	19.2	250	250	124	250	0.7477	0.6223
Hgb	7.37 ± 2.8	4.5	10.1	7.5	4.5	10.1	6.82 ± 1.26	5	10.2	6.85	5.6	7.3	0.7692	0.6809
Plt	105.33 ± 34.95	76	144	96	76	144	99.65 ± 33.7	39	183	88	78	119	0.7887	0.8190
Hct	21.8 ± 8.02	13.5	29.5	22.4	13.5	29.5	20.78 ± 3.74	15	30.6	20.7	17.3	22.5	0.8473	0.7492

AP—antiplasmin; APTT—activated partial thromboplastin time; AT—antithrombin; DD—D-dimer; fbg—fibrinogen; FDP—fibrin/fibrinogen degradation product; FMC—fibrin monomer complex; Hgb—hemoglobin; Hct—hematocrit; PIC—α_2_ plasmin inhibitor/plasmin complex; Plt—platelet; PT—prothrombin time; PT-INR—prothrombin time–international normalized ratio; SD—standard deviation; TAT—thrombin antithrombin III complex.

**Table 2:** Regression functions from fibrinogen to Hgb/fbg and PT-sec and from fibrin/fibrinogen degradation products to D-dimer while avoiding multicollinearity.

Formula	Estimate ± SE	*p*-Value	AIC	R-Squared
Hgb/fbg = *β*_0_ + *β*_1_ fbg	*β*_0_, 174.41 ± 15.277; *β*_1_, −0.898 ± 0.126	*β*_0_, 1.81 × 10^−10^; *β*_1_, 5.31 × 10^−7^	213.79	0.7057
PT-sec = *β*_0_ + *β*_1_ fbg	*β*_0_, 19.681 ± 1.118; *β*_1_, −0.048 ± 0.009	*β*_0_, 4.80 × 10^−14^; *β*_1_, 3.30 × 10^−5^	93.54	0.5679
D-dimer = *β*_0_ + *β*_1_ FDP	*β*_0_, −9.535 ± 20.235; *β*_1_, 3.4946 ± 0.492	*β*_0_, 0.642; *β*_1_; 5.24 × 10^−7^	265.83	0.7061

AIC—Akaike’s information criterion; fbg—fibrinogen; FDP—fibrin/fibrinogen degradation product; Hgb—hemoglobin; PT—prothrombin time; SE—standard error.

**Table 3:** Comparison of the performance of the classifiers on the boundaries in the fibrinogen and fibrin/fibrinogen degradation product planes of hematuria occurrence in parturient hemorrhage for different machine learning methods. All methods were highly accurate (>0.91). All methods demonstrated high AUC values of >0.95. Class mean class entropy was smaller for the logistic regression, naïve Bayes, and neural network methods.

	Accuracy ± SD	AUC	Class Mean Class Entropy	Cohen’s Kappa	F1 Score	PPV, Precision	Sensitivity, Recall	Specificity
Logistic regression	1.000 ± 0.22	1.000	1.417 × 10^−4^	1.000	1.000	1.000	1.000	1.000
Naïve Bayes	0.9565 ± 0.04	0.9583	5.097 × 10^−5^	0.8321	0.8571	0.7500	1.000	0.950
Nearest neighbors	1.000 ± 0.22	1.000	0.3285	1.000	1.000	1.000	1.000	1.000
Neural network	1.000 ± 0.22	1.000	2.253 × 10^−5^	1.000	1.000	1.000	1.000	1.000
Random forest	0.9130 ± 0.06	1.000	0.2407	0.7013	0.7500	0.6000	1.000	0.9000
Support vector machine	0.9565 ± 0.04	1.000	0.4967	0.7767	0.8000	1.000	0.6667	1.000

AUC—area under the characteristic curve; SD—standard deviation.

**Table 4:** Comparison of the performance of classifiers on the boundaries of fibrin/fibrinogen degradation products (FDPs) for hematuria occurrence in parturient hemorrhage by different machine learning methods. Class mean entropy was smaller for the logistic regression, naïve Bayes, and neural network methods. The classifiers by the logistic regression, naïve Bayes, and neural network methods were equally superior.

	FDP Criteria (mg/dL)	Accuracy ± SD	AUC	Class Mean Entropy	Cohen’s Kappa	F1 Score	PPV, Precision	Sensitivity, Recall	Specificity
Logistic regression	84.96	1.000 ± 0.22	1.000	1.417 × 10^−5^	1.000	1.000	1.000	1.000	1.000
Naïve Bayes	86.94	0.9565 ± 0.04	0.958	5.097 × 10^−5^	0.832	0.857	0.750	1.000	0.950
Nearest neighbors	73.38	1.000 ± 0.22	1.000	0.3285	1.000	1.000	1.000	1.000	1.000
Neural network	92.23	1.000 ± 0.22	1.000	3.569 × 10^−4^	1.000	1.000	1.000	1.000	1.000
Random forest	101.16	0.9130 ± 0.06	1.000	0.2407	0.701	0.750	0.600	1.000	0.900
Support vector machine	79.67	0.9565 ± 0.04	1.000	0.4967	0.7767	0.800	1.000	0.6667	1.000

AUC—area under the characteristic curve; SD—standard deviation.

## Data Availability

The datasets generated during this study are available from the corresponding author upon reasonable request.
